# Dysregulated homeostatic pathways in sarcopenia among frail older adults

**DOI:** 10.1111/acel.12842

**Published:** 2018-10-09

**Authors:** Tze Pin Ng, Yanxia Lu, Robin Wai Mun Choo, Crystal Tze Ying Tan, Ma Shwe Z. Nyunt, Qi Gao, Esther Wing Hei Mok, Anis Larbi

**Affiliations:** ^1^ Gerontology Research Programme, Department of Psychological Medicine, Yong Loo Lin School of Medicine National University of Singapore Singapore Singapore; ^2^ Singapore Immunology Network (SIgN) Agency for Science, Technology and Research (A*STAR) Singapore Singapore; ^3^ Geriatric Education and Research Institute, Ministry of Health Singapore Singapore

**Keywords:** anemia, blood biomarkers, cortisol, cytokines, DHEAS, glomerular, inflammation, insulin, leptin, muscle mass and function, testosterone

## Abstract

Sarcopenia, a core feature of the physical frailty syndrome, is characterized by multisystem physiological dysregulation. No study has explored qualitatively the hierarchical network of relationships among different dysregulated pathways involved in the pathogenesis of sarcopenia. We used 40 blood biomarkers belonging to community‐dwelling prefrail and frail older persons to derive measures of multiple physiological pathways, and structural equation modeling to generate path network models of the multisystem physiological dysregulations associated with muscle mass and function (MMF). Insulin–leptin signaling and energy regulation, anabolic sex steroid regulation (testosterone, leptin), and tissue oxygenation (hemoglobin, red cell count) appear to be primary mediating factors exerting direct influences on MMF. There was additionally secondary mediatory involvement of myocyte‐ and adipocyte‐derived cytokines, hypothalamic pituitary adrenal (HPA) stress hormones (cortisol, DHEAS), glomerular function, and immune cell regulatory and inflammatory cytokines and glycoproteins. We conclude that within a hierarchical network of multisystem physiological dysregulations in sarcopenia, dysregulated anabolic and catabolic pathways via sex steroids and insulin–leptin dual signaling and tissue hypoxemia are primary physiological dysregulations responsible for sarcopenia and frailty.

## 1 INTRODUCTION

Studies suggest that multiple cellular, hormonal, metabolic, and molecular mechanisms underlie the development of sarcopenia in animal models, including impaired insulin signaling, imbalanced anabolic and catabolic energy metabolism, upregulation of catabolic cytokine expression, immune dysfunction, systemic inflammation, and increased oxidative stress (A Sayer, Stewart, Patel, & Cooper, 2010). Testosterone is well known to increase muscle mass by increasing the rate of muscle protein synthesis partly via stimulating IGF‐1 expression (Mudali & Dobs, 2004). Insulin appears to play a crucial role in maintaining the rate of muscle protein synthesis and breakdown, both in health and disease (Abdulla, Smith, Atherton, & Idris, 2016). Although the exact mechanism is not fully understood, as a key anabolic hormone, insulin stimulates muscle growth via secreting IGF‐1 followed by activation of the PI3K and Akt (also known as protein kinase B) signaling pathways via mammalian target of rapamycin (mTOR) that controls protein synthesis and the forkhead box O (FOXO) that controls protein degradation. Current evidence suggests that the anabolic effect of insulin is driven by attenuating muscle protein breakdown rather than increasing muscle protein synthesis.

These and other hormonal and neuroendocrine markers of anabolic and catabolic imbalance associated with sarcopenia and physical frailty in humans have been investigated individually in small numbers of observational studies. Circulating insulin (Barzilay et al., 2007; Cleasby, Jamieson, & Atherton, 2016), testosterone (Auyeung et al., 2011), insulin‐like growth factor‐1 (IGF‐1; Gielen et al., 2015), and dehydroepiandrosterone sulfate (DHEAS; Tajar et al. (2011)) have been interrogated in a majority of studies mostly in isolation and with conflicting results. In contrast, several studies, such as the InCHIANTI Study, the WHAS I and II, and the MSSA (Fried et al., 2009; Gruenewald, Seeman, Karlamangla, & Sarkisian, 2009; Maggio et al., 2010), have found that an increased number of multiple hormonal biomarker abnormalities strongly predict physical frailty when individual biomarker abnormalities did not. Determining the effect of a single physiological derangement in sarcopenia is complicated by the effects of intermediary changes in the complex network of physiological pathways that are deranged in sarcopenia.

Other individual biomarkers of physiological dysregulations that have been interrogated in human observational studies include pro‐inflammatory cytokines such as C‐reactive proteins (CRPs), interleukin‐6 (IL‐6) and tumor necrosis factor‐α (TNF‐α) (Hubbard, O'Mahony, Savva, Calver, & Woodhouse, 2009; Westbury et al., 2017) and inflammatory glycoproteins in the coagulation cascade such as factor VIII, D‐dimer, tissue plasminogen activator (t‐PAS; Walston et al., 2002), cellular and circulating markers of innate immunity (Leng et al., 2009), oxidative stress (Serviddio et al., 2009; Wu, Shiesh, Kuo, & Lin, 2009), and anemia (Hirani et al., 2016), and low kidney function (Wang & Mitch, 2014). Also, the results tend to be inconsistent for individual biomarkers, whereas the number of biomarker impairments across multiple physiological systems strongly predicted physical frailty (Fried et al., 2009; Gruenewald et al., 2009).

Because sarcopenia involves physiological dysregulation within and across multiple systems, its pathophysiology could be better understood when combinations of biomarkers and physiological impairments are investigated together rather than singly. Thus far, no study has explored the qualitative hierarchy of relationships within a network of different dysregulated pathways involved in the pathogenesis of sarcopenia.

In this study, we used archival baseline blood specimens collected in a previously reported study of prefrail and frail older persons in the Frailty Intervention Trial in Singapore (Ng et al., 2015), to analyze known and potential blood biomarkers of sarcopenia and frailty. We chose 40 analytes of hormones, cytokines, metabolites, and other biomarkers that were identified from literature search to be involved with homeostatic processes associated with muscle mass and function and dysregulated in sarcopenia and frailty. The study subjects’ mean age was 69.3 (*SD*: 4.2) years; they included 34 males and 63 females and 18 with known history of diabetes. The mean appendicular mean mass, knee strength, and gait speed are shown in Supporting information Table S1. Using a guided supervision approach to principal component analyses of the 40 blood biomarker measurements, we derived various measures of physiological and molecular activity across multiple important regulatory systems (Table 1).

**Table 1:** Principal component analysis of biological function factors represented by blood biomarkers

Factor and Item Variable	Loading	
Muscle mass and function		
ALM/Ht^2^	0.703	
Knee strength	0.894	
Gait speed	0.487	
Immune cell and inflammation homeostasis		
Soluble tumor necrosis factor receptor 2	0.873	
Soluble tumor necrosis factor receptor 1	0.842	
TNF‐α	0.576	
CRP	0.574	
Interleukin‐6 (IL‐6)	0.510	
Interferon‐gamma‐inducible protein 10 kDa (IP‐10)	0.384	
Soluble glycoprotein 130 (sgp130)	0.330	
Acute phase protein immune stress response‐I		
B‐2‐microglobulin	0.830	
Ferritin	0.808	
Albumin	−0.481	
Acute phase protein immune stress response‐II		
Soluble glycoprotein 130	0.742	
Haptoglobin	0.645	
Transferrin	−0.606	
Acute phase protein immune stress response‐III		
Transferrin	0.688	
Albumin	0.560	
D‐Dimer	−0.655	
Acute phase cytokine immune stress response‐IV		
Interleukin‐6	0.974	
TNF‐α	0.974	
Acute phase response/iron metabolism		
Transferrin	0.736	
Ferritin	−0.736	
Cellular immune activation and anti‐oxidation		
Oxidized glutathione		0.846
Neopterin		0.846
Oxidative stress (lipid peroxidation)		
Reduced glutathione		0.714
4‐hydroxynonenal (4HNE)		−0.714
4‐Carbon methylation		
Homocysteine		−0.849
Folate		0.613
B12		0.547
Insulin signaling and energy homeostasis		
C‐peptide		0.880
Insulin		0.811
Leptin		0.742
Anabolic sex steroid homeostasis		
Free testosterone		0.806
Leptin		−0.806
Energy homeostasis		
Active ghrelin		0.762
IGF‐1		0.762
HPA stress response		
Cortisol		0.873
Dehydroepiandrosterone sulfate		−0.873
Myocyte protein signaling		
Irisin		0.707
Myostatin		−0.707
Adipocyte protein signaling		
Adiponectin		0.873
Obestatin		0.873
Oxygen transport and delivery		
Hemoglobin		0.915
Red blood cell count		0.915
Glomerular function		
eGFR (cystatin)		0.806
eGFR (creatinine)		0.806
Thyroid‐mediated catabolic homeostasis		
Thyroid‐stimulating hormone		0.719
Triiodothyronine		0.719
Mineral metabolism and bone modeling		
Osteopontin		0.708
Parathyroid hormone		0.708

Notes

Loadings shown in the table are factor loadings/component coefficients in principal component analysis. The factor loadings are the correlation coefficients between the variables and factors/components. The factors were represented by variables with the corresponding highest loadings among all the 40 included variables in the analysis.

Preliminary analyses revealed significant univariate associations (*p* < 0.05) with muscle mass and function (MMF) score individually for creatinine, B12, folate, homocysteine, oxidized glutathione, adiponectin, ferritin, amyloid protein, insulin, C‐peptide, free testosterone, active ghrelin, rbc count, and hemoglobin (Supporting information Table S2). In a backward selection final model including age and sex as covariates, independent significant blood biomarkers among them simultaneously (*p* < 0.05) predicting MMF score were C‐peptide, active ghrelin, free testosterone, parathyroid hormone, reduced glutathione, 4‐hydroxynonenal, cystatin C, rbc count, sTNF‐R1, ferritin, and myostatin, retained at *p* = 0.081 (Supporting information Table S3).

**Figure 1:**
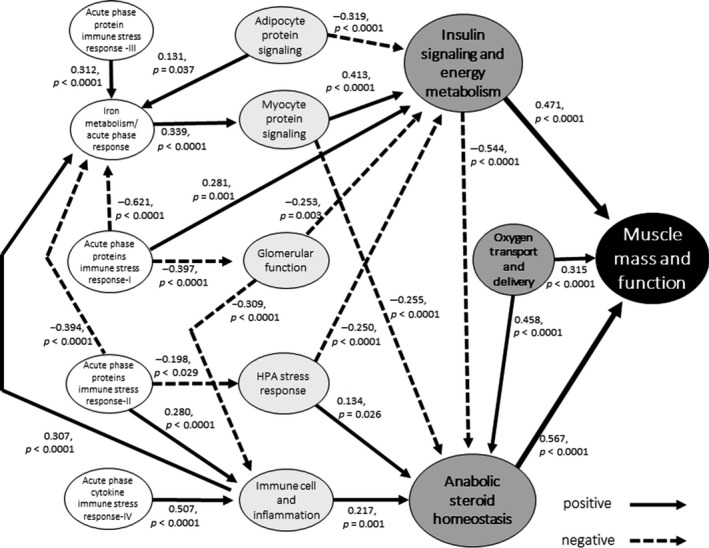
Path analysis of blood biomarkers and muscle mass and function

We analyzed the inter‐relationships of these blood biomarker measures among themselves and with MMF and generated path network models of the multisystem physiological dysregulations involved in sarcopenia (Figure 1) using structural equation modeling (model fit indices indicating acceptable model fit to the data are shown in Supporting information Table S4). The models revealed three tiers of biomarker relationships with MMF as shown in Supporting information Table S5 (Path Model Results) and Figure 1 (Path Model Diagram). (More information on the statistical analysis procedures may be obtained in writing to the corresponding author.).

The first tier of primary relationships with MMF, indicated by positive biomarker associations with (a) anabolic sex steroid regulation (*β* = 0.567, *p* < 0.001), (b) insulin signaling and energy metabolism (*β* = 0.315, *p* < 0.001), and (c) oxygen transport and delivery (*β* = 0.471, *p* < 0.001), suggests that these are primary processes involved in muscle growth and function that are dysregulated in sarcopenia.

1. *Insulin signaling and energy metabolism *(ISEM: denoted by insulin, C‐peptide, leptin). Based on present evidence, it remains unclear whether increased insulin resistance or reduced endogenous insulin secretion (or both) is underlying mechanisms in sarcopenia and frailty (Cleasby et al., 2016; Tanaka, Kanazawa, & Sugimoto, 2015). Our result shows that low fasting plasma insulin and C‐peptide (a better measure of portal insulin secretion) levels are associated with low muscle mass and function and suggests that in this Asian group, endogenous reduction of insulin level and dysregulated insulin signaling activity in older individuals (with and without diabetes) diminish the anabolic capacity of insulin to alleviate muscle protein breakdown in skeletal muscles. As expected, basal plasma leptin is increased together with increased insulin, and higher concentrations of both hormones appear to be directly related to greater muscle mass and function. Conversely, in physically frail individuals with low body mass, the presence of decreased levels of leptin (known to be associated with weight loss) may reflect a state of persistent energy deprivation that characterizes physical frailty.
2. *Anabolic sex steroid regulation (ASSR: free testosterone, leptin).* Next to insulin–leptin signaling and energy metabolism, ASSR appears to occupy a co‐pivotal role in the multiple hormonal dysregulation in sarcopenia. ASSR is inversely linked to ISEM, reflecting what is known about their complementary actions in protein synthesis and muscle accretion (Mudali & Dobs, 2004). Taken together, dysregulated anabolic and catabolic pathways via insulin signaling and energy metabolism and anabolic sex steroid regulation appear to be primary pathophysiological mechanisms directly related to the development of sarcopenia.
3. *Oxygen transport and delivery (hemoglobin, rbc* *count).* This index of tissue oxygenation is also directly and indirectly (via its link to ASSR) associated with muscle mass and function. Reduced tissue oxygen delivery with anemia, hypoxia, or ischemia in skeletal muscle has been shown to impair muscle strength and performance (Dodd, Powers, Brooks, & Crawford, 1993). In line with this, there are reports of the association of anemia with physical frailty (Hirani et al., 2016; Roy, 2011), which may be due to chronic inflammation related to aging, physical inactivity, chronic diseases, or nutritional deficiency.

There appears to be a second tier of five intermediary physiological processes that are indirectly linked to muscle mass and function via their positive or inverse associations with ISEM or ASSR.

1. *Myocyte protein signaling (irisin and myostatin)*. Its positive link to ISEM is consistent with their known effects on activation of the PI3K‐Akt‐mTOR pathway and the FOXO pathway mentioned above. Myostatin, a negative regulator of skeletal muscle growth, is known to negatively regulate the activity of the Akt pathways, which promotes protein synthesis and increases the activity of the ubiquitin–proteasome system to induce atrophy (Rodriguez et al., 2014). On the other hand, the negative relationship between myocyte signaling proteins and ASSR is consistent with studies showing androgens to have strong negative impact on myostatin expression (Mendler, Baka, Kovács‐Simon, & Dux, 2007).
2. *Adipocyte protein signaling (adiponectin* *and obestatin). *This is negatively linked to ISEM, consistent with the known influence of adipokines on beta cell function through enhancing or inhibiting insulin release (Cantley, 2014), pointing to the dysregulated production of adipokines in sarcopenia associated with obesity.
3. *HPA* *stress response (cortisol and DHEAS). *Studies of men with high variability of cortisol secretion (indicating normal regulation of the HPA) have shown that cortisol secretion is positively associated with blood testosterone and fasting insulin levels, whereas in men with low variability of diurnal cortisol secretion (indicating abnormal regulation of the HPA axis), cortisol secretion shows strong negative relationships with testosterone and insulin (Rosmond, Dallman, & Björntorp, 1998). In line with this, our results indeed showed that cortisol was associated negatively with fasting insulin and positively with testosterone levels among these physically prefrail and frail older persons, possibly indicating a more sensitive effect on insulin–leptin axis dysregulation than anabolic testosterone dysregulation.
4. *Glomerular function. *Muscle wasting in kidney disease (from mild to severe) is well established through catabolic pathways including activation of the ubiquitin–proteasome system (UPS), caspase‐3, lysosomes, and myostatin (Wang & Mitch, 2014).
5. *Immune cell and inflammation homeostasis (TNF‐*α*, sTNFR‐1, sTNFR‐2, IL‐6, CRP, IP‐10, sgp130). *Age‐associated decline in immune cell function (such as neutrophil migration) and chronic inflammation and dysregulation of the phosphoinositide 3‐kinase (PI3K)‐Akt pathway in neutrophils are strongly believed to contribute pathogenically to sarcopenia and frailty, but a direct causative role is not well established (Wilson, Jackson, Sapey, & Lord, 2017). In particular, it showed a positive association with ASR in this study, but this is not consonant with prior experimental findings that IL‐6 and TNF‐α inhibit testosterone secretion via central hypothalamic–pituitary and peripheral gonadal influences, and observations in men that serum levels of testosterone are inversely related to soluble IL‐6 receptor (sIL‐6R) and the effect of testosterone supplementation in reducing inflammatory markers in hypogonadal men (Maggio et al., 2005).

In connection to the above, the findings also reveal an associated series of innate immune system response processes represented almost entirely of positive or negative acute phase proteins (plasma transport, coagulation, and other inflammatory glycoproteins), which have also been reported in human observational studies to be associated with sarcopenia and frailty (Darvin et al., 2014; Reiner et al., 2009; Shamsi et al., 2012). Their observed corresponding positive and inverse relationships with adipokine signaling, myokine signaling, insulin signaling, HPA stress response, and glomerular function in this study have not been reported in the literature and are difficult to explain, and may possibly be epiphenomenon.

In summary, this study used a large number of specific blood biomarkers carefully chosen for their potential involvements in the multisystem physiological dysregulations in sarcopenia. Nevertheless, there are potential limitations in our ability to employ effectively all possible relevant biomarkers for inclusion in the study. Our portrayed network of physiological dysregulations may not be all inclusive, and there may be additional pathways and networks that may not be represented given the range biomarkers and the analytical strategy we used. Also, there may be more than one way of interpreting and depicting the multiple physiological pathways associated with a single blood biomarker or a combination of biomarkers. Future work may further refine our understanding of this network relationship. As it stands, however, this network model reveals new insights into the hierarchy of relationships among physiological systems, which are dysregulated in sarcopenia. This has important clinical implications in facilitating the identification of strategic approaches in modifying the key pathophysiological processes involved in sarcopenia and physical frailty for potential therapeutic and preventive interventions.

## CONFLICT OF INTEREST

None reported.

## AUTHOR CONTRIBUTION

TPN formulated the hypothesis, performed literature review, designed the study, analyzed and reviewed the data, and drafted and reviewed the manuscript. RWMC performed the statistical analyses. CTYT and EWHM performed the laboratory analyses. YL, CTYT, MSZN, QG, EWHM, and AL contributed to the review of the literature, study design, and data collection, and review of the data and manuscript drafts.
