# Supplementary material for: Dysregulated homeostatic pathways in sarcopenia among frail older adults
Source: Aging Cell. 2018 Oct 9;17(6):e12842. doi: 10.1111/acel.12842 (PMC6260914; doi:10.1111/acel.12842)
Supplement: Supplementary file 1 [file ACEL-17-e12842-s001.docx]

**Supplementary**

**Experimental procedures**

Details of the Singapore Frailty Intervention Trial (S-FIT) (clinicaltrial.gov identifier NCT00973258) have been described in a previous publication (Ng *et al.* 2015). The study was approved by the National Health Group (NHG) Domain Specific Review Board in Singapore. The study participants were community-living older adults aged 65 years and above who were pre-frail and frail with scores of 1 to 2 (pre-frailty) and 3 to 5 (frailty) based on the widely accepted criteria defining the physical frailty phenotype (Fried *et al.* 2001): unintentional weight loss, slowness, weakness, exhaustion and low activity. They were able to ambulate without personal assistance and living at home and excluded those who had dementia or significant cognitive impairment (Mini-mental State Examination score ≤ 23), major depression, severe audio-visual impairment, degenerative neurologic disease, and terminal illness with life expectancy less than 12 months. In this randomized control trial, eligible and consenting participants were allocated into one of 5 interventions of 24 weeks duration each: nutritional supplementation, cognitive training, physical training, combination treatment and usual care control. Details of the interventions are reported in a previous paper. Measurements of domains of physical, mood and cognitive functions were made pre-intervention at 0 month (0M), and post-intervention at 3 months (3M), 6 months (6M) and 12 months (12M).

The present study analyzed data of measurements of sarcopenia and related blood biomarkers in archival serum specimens collected at baseline.

*Muscle mass and function*

1. *Lean body mass* was measured by dual energy X-ray absorptiometry (DXA) using the Hologic® densitometer. Tests were performed by operators in accordance with the manufacturer’s protocol with the participant in the supine position. Appendicular lean mass (ALM) was calculated by summing lean mass (kg) of the two upper limbs and two lower limbs, with the adjustment of limb cut lines according to specific anatomical landmarks (Heymsfield *et al.* 1990). The estimated ALM was corrected for total body mass by dividing it with the square of the height (ALM/ht^2^).
2. *Lower limb knee extension strength* was measured using Lord’s trap and strain gauge assembly component of the Physiological Profile Assessment (APA) (Lord *et al.* 2003) using the average value from 3 trials on the dominant knee.
3. *Gait speed.* The 6-meter fast gait speed test was performed following standardized procedures. Briefly, participants completed 6-meter walking as fast as possible, and the time in seconds taken to complete 6 metres for 2 trials was averaged.

*Serum biomarkers*

Archival serum specimens were thawed and quantitative assays were performed for 40 analytes of hormones, cytokines, metabolites and other biomarkers that were identified from literature search to be involved with homeostatic processes associated with sarcopenia and frailty. The list of blood biomarkers are shown in **Table 1.**

Ferritin, free testosterone, TSH, cortisol, T3, DHEA-s, transferin, obestatin, D-dimer, GSH, GSSG, HNE protein plasma adducts, myostatin, neopterin, irisin, sIL-2R, adiponection and CRP were measured via the enzyme-linked immunosorbent assay. Serum samples were added to wells of ready-coated plates coated with antibodies specific to the marker of interest. The bound markers were then incubated with detection antibodies. The absorbance resulting from the enzymatic reaction from the enzyme on the detection antibodies and added substrate was measured using a microplate reader. Other markers (insulin, leptin, C-peptide, ghrelin active, haptoglobin, serum amyloid P (SAP), sgp130, sTNFR1, sTNFR2, IP-10, osteopontin, PTH, cystatin C, beta-2-microglobulin, IGF-1) were measured using the Luminex assay (Merck Millipore). Samples were incubated with beads, each uniquely fluorescent and conjugated to specific antibodies against the marker of interest. Detection antibodies were then added to the complex and the fluorescence was measured using a Luminex analyzer. Albumin and creatinine were measured using standard clnical chemistry methods at NUH Referral Laboratories. TNF-a, IL-6, folate and vitamin B12 were run on Immulite analyser from Siemens Healthcare. Homocysteine was run on Axysm analyser from Abbott Diagnostics.

Statistical analyses

We analyzed a complete set of available data of all muscle and blood biomarkers belonging to 97 subjects. A global z-score of muscle mass and function (MMF) was estimated by the principal component analysis (PCA) score derived from ALM/ht^2^, knee extension strength and gait speed. The standardized regression factor scores for selected principal components as statistical outputs were saved for each subject, and summed as a z-score for each subject after weighing respective variances explained. Higher z-values of MMF denote higher muscle mass and function level among these pre-frail and frail subjects. Similarly, composite z-scores derived from principal component analysis were created for measures of two or more blood biomarkers to represent various homeostatic regulatory pathways involved in sarcopenia. These representations were based on selecting biomarkers known to be critically involved in established biological pathways such as insulin signaling and energy metabolism, testosterone-mediated anabolic homeostasis, thyroid-mediated catabolic homeostasis, acute phase stress response, immune cell and inflammation homeostasis, myocyte and adipocyte protein signaling, hypothalamic–pituitary–adrenal (HPA) stress response, cellular immune activation and anti-oxidation, mineral metabolism and bone modeling, glomerular function, oxidative stress, iron transport and metabolism, 4-carbon methylation, and oxygen transport and delivery **(Table 1)**. As shown in **Table 1**, MMF and homeostatic factors derived from principal component analyses were all single component factors with strong loadings.

*Structural equation modeling and path network analyses.* Blood biomarker factor scores and the muscle mass and function factor score were analyzed with Pearson's pairwise correlations in a preliminary analysis. In the path model, the muscle mass and function factor score was regressed on the first set of biomarker factors which were significantly correlated in the preliminary correlation analysis. The first tier of biomarker factors were regressed on its respective second tier of correlated biomarker factors which in turn were regressed on a third tier of correlated biomarker factors. Lastly, additional regression paths were added among biomarker factors using modification indices (with a minimum cutoff of χ^2^ of 3.84). The final path model is shown in Figure 1 (Path Model Diagram). The Pearson's pairwise correlations were performed by Stata/SE 12.0 and the path model was performed by Mplus 7.11. Likelihood χ^2^ test of model fit and four other approximate fit indexes (RMSEA, CFI, TFI and SRMR) produced from Mplus were evaluated to determine plausible model fit. Individual path coefficients in the model were tested statistical significance given an acceptable model fit. All path coefficients were reported as standardized betas (β).

**References**

Fried LP, Tangen CM, Walston J, Newman AB, Hirsch C, Gottdiener J, Seeman T, Tracy R, Kop WJ, Burke G, McBurnie MA, Cardiovascular Health Study Collaborative Research G (2001). Frailty in older adults: evidence for a phenotype. *The journals of gerontology. Series A, Biological sciences and medical sciences*. **56**, M146-156.

Heymsfield SB, Smith R, Aulet M, Bensen B, Lichtman S, Wang J, Pierson RN, Jr. (1990). Appendicular skeletal muscle mass: measurement by dual-photon absorptiometry. *The American journal of clinical nutrition*. **52**, 214-218.

Lord SR, Menz HB, Tiedemann A (2003). A physiological profile approach to falls risk assessment and prevention. *Physical therapy*. **83**, 237-252.

Ng TP, Feng L, Nyunt MS, Feng L, Niti M, Tan BY, Chan G, Khoo SA, Chan SM, Yap P, Yap KB (2015). Nutritional, Physical, Cognitive, and Combination Interventions and Frailty Reversal Among Older Adults: A Randomized Controlled Trial. *The American journal of medicine*. **128**, 1225-1236.e1221.

von Haehling S, Morley JE, Coats AJS, Anker SD (2017). Ethical guidelines for publishing in the journal of cachexia, sarcopenia and muscle: update 2017. *J Cachexia Sarcopenia Muscle*. **8**, 1081-1083.

**Supplementary Table 1. Muscle mass and function of pre-frail and frail study subjects**

|  | Whole sample  (N=97) | |  | Males  (N=34) | |  | Females  (N=63) | |
| --- | --- | --- | --- | --- | --- | --- | --- | --- |
|  |  |  |  |  |  |  |  |  |
| Appendicular muscle mass, kg/m^2^ | 6.1 | ± 1.1 |  | 7.2 | ± 0.8 |  | 5.6 | ± 0.8 |
| Gait speed, m/s | 0.95 | ± 0.23 |  | 0.98 | ± 0.23 |  | 0.93 | ± 0.22 |
| Knee strength, kg | 14.7 | ± 5.5 |  | 18.9 | ± 6.3 |  | 12.4 | ± 3.3 |
| Low muscle mass | 38 | (39.2) |  | 11 | (32.4) |  | 27 | (43.9) |
| Low knee strength | 89 | (91.8) |  | 30 | (88.2) |  | 59 | (93.7) |
| Slow gait | 27 | (27.8) |  | 7 | (20.6) |  | 20 | (31.7) |
| Sarcopenia | 35 | 36.1 |  | 9 | (26.5) |  | 26 | (41.3) |
|  |  |  |  |  |  |  |  |  |
| Figures are mean ± SD or N (%)  Low knee strength: knee strength <26 kg (males) or 18 kg (females)  Slow gait: gait speed < 0.8 m/s  Sarcopenia: Low muscle mass and (low knee strength or slow gait) | | | | | | | | |

| **Supplementary Table 2. Univariate associations of individual biomarkers with muscle mass and function factor score** | | | | | |
| --- | --- | --- | --- | --- | --- |
|  | | | | | |
|  | Unstandardized | | Standardized |  |  |
|  | b | Std. Error | Beta | t | Sig. |
| Albumin | 0.044 | 0.048 | 0.095 | 0.930 | 0.355 |
| Creatinine | 0.016 | 0.004 | 0.368 | 3.856 | <0.001 |
| B12 | -0.001 | 0.000 | -0.221 | -2.212 | 0.029 |
| Folate | -0.034 | 0.019 | -0.176 | -1.746 | 0.084 |
| Homocysteine | 0.039 | 0.018 | 0.217 | 2.172 | 0.032 |
| Neopterin | 0.024 | 0.016 | 0.150 | 1.478 | 0.143 |
| Reduced Glutathione | 7.370E-5 | 0.001 | 0.011 | 0.109 | 0.914 |
| Oxidized Glutathione | 0.010 | 0.005 | 0.213 | 2.126 | 0.036 |
| 4-hydroxynonenal | -0.138 | 0.076 | -0.183 | -1.810 | 0.073 |
| Irisin | -0.003 | 0.002 | -0.125 | -1.232 | 0.221 |
| Myostatin | 0.006 | 0.005 | 0.123 | 1.213 | 0.228 |
| Adiponectin | -0.009 | 0.004 | -0.230 | -2.305 | 0.023 |
| Obestatin | -0.001 | 0.001 | -0.146 | -1.441 | 0.153 |
| Transferrin | -0.002 | 0.001 | -0.117 | -1.149 | 0.254 |
| Ferritin | 0.003 | 0.001 | 0.267 | 2.697 | 0.008 |
| Triiodothyronine | -0.050 | 0.287 | -0.018 | -0.174 | 0.862 |
| Thyroid-Stimulating Hormone | -0.011 | 0.080 | -0.014 | -0.134 | 0.893 |
| Dehydroepiandrosterone-sulfate | 0.001 | 0.001 | 0.076 | 0.745 | 0.458 |
| Cortisol | 0.025 | 0.034 | 0.077 | 0.755 | 0.452 |
| Amyloid P | 0.008 | 0.003 | 0.263 | 2.655 | 0.009 |
| Cystatin-C | 0.009 | 0.009 | 0.104 | 1.017 | 0.312 |
| D-Dimer | -0.002 | 0.002 | -0.144 | -1.422 | 0.158 |
| Haptoglobin | 0.016 | 0.037 | 0.045 | 0.441 | 0.660 |
| β-2-microglobulin | 0.003 | 0.003 | 0.104 | 1.021 | 0.310 |
| Insulin | 0.001 | 0.000 | 0.316 | 3.242 | 0.002 |
| C-peptide | 0.376 | 0.109 | 0.335 | 3.462 | 0.001 |
| IGF-1 | 0.131 | 0.129 | 0.103 | 1.008 | 0.316 |
| Free Testosterone | 0.141 | 0.019 | 0.605 | 7.415 | <0.001 |
| Leptin | -0.007 | 0.010 | -0.067 | -0.650 | 0.517 |
| Active Ghrelin | 0.010 | 0.021 | 0.047 | 0.460 | 0.647 |
| CRP | -1.011-6 | 0.000 | -0.014 | -0.134 | 0.894 |
| TNF-a | -0.006 | 0.008 | -0.082 | -0.801 | 0.425 |
| Interleukin 6 | -0.001 | 0.008 | -0.011 | -0.111 | 0.912 |
| Interleukin 2-Receptor | -0.001 | 0.000 | -0.127 | -1.246 | 0.216 |
| Soluble Tumor Necrosis Factor Receptor 1 | 0.033 | 0.098 | 0.035 | 0.339 | 0.735 |
| Soluble Tumor Necrosis Factor Receptor 2 | 0.004 | 0.017 | 0.024 | 0.235 | 0.815 |
| Soluble Glycoprotein 130 | -0.001 | 0.002 | -0.034 | -0.335 | 0.738 |
| Interferon-Gamma Inducible Protein 10 kDa | 0.000 | 0.001 | -0.033 | -0.324 | 0.746 |
| Osteopontin | 0.003 | 0.012 | 0.027 | 0.262 | 0.794 |
| Parathyroid Hormone | 0.003 | 0.004 | 0.076 | 0.740 | 0.461 |
| Red Blood Cell Count | 1.086 | 0.171 | 0.546 | 6.359 | <0.001 |
| Haemoglobin | 0.457 | 0.065 | 0.587 | 7.076 | <0.001 |
| Lymphocytes count | -0.031 | 0.212 | -0.015 | -0.146 | 0.885 |
|  |  |  |  |  |  |

Footnote: p values of significance of biomarker variables are shown with all other biomarker variables present in the model

| **Supplementary Table 3. Multivariate model from final backward selection of independent significant blood biomarkers predicting muscle mass and function z-score (age and sex-adjusted)** | | | | | |
| --- | --- | --- | --- | --- | --- |
| Analyte Biomarkers | Unstandardized | | Standardized |  |  |
|  | b | SE | Beta | t | Sig. |
|  |  |  |  |  |  |
| Age (control variable) | -0.027 | 0.017 | -0.114 | -1.635 | 0.106 |
| Female sex (control variable) | -0.624 | 0.212 | -0.299 | -2.947 | 0.004 |
| Amyloid P component (SAP) | 0.008 | 0.002 | 0.249 | 3.636 | <0.001 |
| C-peptide | 0.268 | 0.076 | 0.239 | 3.547 | 0.001 |
| Active Ghrelin | -0.045 | 0.015 | -0.220 | -2.992 | 0.004 |
| Free Testosterone | 0.077 | 0.023 | 0.329 | 3.371 | 0.001 |
| Parathyroid Hormone | 0.007 | 0.002 | 0.196 | 3.125 | 0.002 |
| Reduced Glutathione | -0.001 | 0.000 | -0.208 | -3.214 | 0.002 |
| 4-hydroxynonenal | -0.120 | 0.045 | -0.159 | -2.699 | 0.008 |
| Cystatin C | 0.017 | 0.006 | 0.190 | 2.728 | 0.008 |
| Red Blood Cell Count | 0.428 | 0.147 | 0.216 | 2.918 | 0.005 |
| Soluble Tumor Necrosis Factor Receptor 1 | -0.137 | 0.067 | -0.144 | -2.034 | 0.045 |
| Ferritin | 0.002 | 0.001 | 0.171 | 2.298 | 0.024 |
| Myostatin | -0.006 | 0.003 | -0.122 | -1.749 | 0.084 |
|  |  |  |  |  |  |

**Supplementary Table 4. Model fit of Structural Equation Modelling**

| **Model fit information** | |  |  |
| --- | --- | --- | --- |
|  |  |  |  |
| Number of Free Parameters | | 39 |  |
|  |  |  |  |
| Log likelihood | |  |  |
|  | H0 Value | -686.903 |  |
|  | H1 Value | -658.74 |  |
|  |  |  |  |
| Information Criteria | |  |  |
|  | Akaike (AIC) | 1451.806 |  |
|  | Bayesian (BIC) | 1551.407 |  |
|  | Sample-Size Adjusted BIC (n* = (n + 2) / 24) | 1428.275 |  |
|  |  |  |  |
| Chi-Square Test of Model Fit | |  |  |
|  | Value | 56.325 |  |
|  | Degrees of Freedom | 45 |  |
|  | P-Value | 0.1200 |  |
|  |  |  |  |
| RMSEA (Root Mean Square Error Of Approximation) | |  |  |
|  | Estimate | 0.051 |  |
|  | 90 Percent C.I. | 0.000 | 0.090 |
|  | Probability RMSEA <= 0.05 | 0.451 |  |
|  |  |  |  |
| CFI/TLI | |  |  |
|  | CFI | 0.976 |  |
|  | TLI | 0.962 |  |
|  |  |  |  |
| Chi-Square Test of Model Fit for the Baseline Model | |  |  |
|  | Value | 533.903 |  |
|  | Degrees of Freedom | 70 |  |
|  | P-Value | 0 |  |
|  |  |  |  |
| SRMR (Standardized Root Mean Square Residual) | | 0.052 |  |
|  |  |  |  |
|  |  |  |  |

| **Supplementary Table 5. Structural Equation Modelling of Muscle Mass and Functioning** | | |  |  |  |
| --- | --- | --- | --- | --- | --- |
| **Dependent Factors** | **Independent Factors** | **Estimate** | **S.E.** | **Est./S.E.** | **p** |
|  |  |  |  |  |  |
| [ Muscle mass and function ] | [ Anabolic sex steroid homeostasis ] | 0.567 | 0.106 | 5.368 | <0.001 |
|  | [ Insulin signaling and energy metabolism ] | 0.471 | 0.097 | 4.858 | <0.001 |
|  | [ Oxygen transport and delivery ] | 0.315 | 0.088 | 3.564 | <0.001 |
| [ Anabolic steroid homeostasis ] | [ HPA stress response ] | 0.134 | 0.060 | 2.228 | 0.026 |
|  | [ Myocyte protein signaling ] | -0.255 | 0.072 | -3.558 | <0.001 |
|  | [ Insulin signaling and energy metabolism ] | -0.544 | 0.064 | -8.470 | <0.001 |
|  | [ Immune cell and inflammation homeostasis] | 0.217 | 0.063 | 3.446 | 0.001 |
|  | [ Oxygen transport and delivery ] | 0.458 | 0.064 | 7.161 | <0.001 |
| [ Insulin signaling and energy metabolism ] | [ Adipocyte protein signaling ] | -0.319 | 0.074 | -4.308 | <0.001 |
|  | [ HPA stress response ] | -0.250 | 0.078 | -3.186 | 0.001 |
|  | [ Myocyte protein signaling ] | 0.413 | 0.077 | 5.359 | <0.001 |
|  | [ Acute phase protein immune stress response-I ] | 0.281 | 0.086 | 3.274 | 0.001 |
|  | [ Glomerular function ] | -0.253 | 0.082 | -3.064 | 0.002 |
| [ Myocyte protein signaling ] | [ Iron metabolism and acute phase response] | 0.339 | 0.091 | 3.723 | <0.001 |
| [ Iron metabolism and acute phase response ] | [ Adipocyte protein signaling ] | 0.131 | 0.063 | 2.086 | 0.037 |
|  | [ Acute phase proteins immune stress response-III ] | 0.312 | 0.070 | 4.481 | <0.001 |
|  | [ Acute phase proteins immune stress response-II ] | -0.394 | 0.075 | -5.259 | <0.001 |
|  | [ Acute phase proteins immune stress response-I ] | -0.621 | 0.069 | -8.996 | <0.001 |
|  | [ Immune cell and inflammation homeostasis ] | 0.307 | 0.075 | 4.079 | <0.001 |
| [ Glomerular function ] | [ Acute phase proteins immune stress response-II ] | -0.198 | 0.091 | -2.177 | 0.029 |
|  | [ Acute phase proteins immune stress response-I ] | -0.397 | 0.085 | -4.656 | <0.001 |
| [Immune cell and inflammation homeostasis ] | [ Acute phase cytokine immune stress response-IV] | 0.507 | 0.060 | 8.407 | <0.001 |
|  | [ Acute phase response (innate immunity)-II ] | 0.290 | 0.062 | 4.700 | <0.001 |
|  | [ Glomerular function ] | -0.390 | 0.063 | -6.204 | <0.001 |
| Intercepts | [ Muscle mass and function ] | -0.016 | 0.073 | -0.224 | 0.822 |
|  | [ Iron transport and metabolism ] | 0.015 | 0.060 | 0.252 | 0.801 |
|  | [ Anabolic homeostasis ] | -0.032 | 0.059 | -0.539 | 0.590 |
|  | [ Myocyte protein signaling ] | 0.010 | 0.097 | 0.100 | 0.920 |
|  | [ Insulin signaling and energy metabolism ] | -0.039 | 0.076 | -0.521 | 0.603 |
|  | [ Immune cell and inflammation homeostasis ] | 0.153 | 0.064 | 2.405 | 0.016 |
|  | [ Glomerular function ] | -0.018 | 0.093 | -0.200 | 0.842 |
| Residual Variances | [ Muscle mass and function ] | 0.508 | 0.071 | 7.128 | <0.001 |
|  | [ Iron transport and metabolism ] | 0.343 | 0.057 | 6.075 | <0.001 |
|  | [ Anabolic homeostasis ] | 0.330 | 0.050 | 6.539 | <0.001 |
|  | [ Myocyte protein signaling ] | 0.885 | 0.062 | 14.331 | <0.001 |
|  | [ Insulin signaling and energy metabolism ] | 0.541 | 0.069 | 7.840 | <0.001 |
|  | [ Immune cell and inflammation homeostasis ] | 0.338 | 0.055 | 6.141 | <0.001 |
|  | [ Glomerular function ] | 0.810 | 0.072 | 11.189 | <0.001 |
| R-square | [ Muscle mass and function ] | 0.492 | 0.071 | 6.917 | <0.001 |
|  | [ Iron transport and metabolism ] | 0.657 | 0.057 | 11.622 | <0.001 |
|  | [ Anabolic homeostasis ] | 0.670 | 0.050 | 13.287 | <0.001 |
|  | [ Myocyte protein signaling ] | 0.115 | 0.062 | 1.861 | 0.063 |
|  | [ Insulin signaling and energy metabolism ] | 0.459 | 0.069 | 6.647 | <0.001 |
|  | [ Immune cell and inflammation homeostasis ] | 0.662 | 0.055 | 12.024 | <0.001 |
|  | [ Glomerular function ] | 0.190 | 0.072 | 2.620 | 0.009 |
|  |  |  |  |  |  |
